# High Incidence of Acute Liver Failure among Patients in Egypt Coinfected with Hepatitis A and Hepatitis E Viruses

**DOI:** 10.3390/microorganisms11122898

**Published:** 2023-11-30

**Authors:** Mohamed A. El-Mokhtar, Amal A. Elkhawaga, Mona Sedky Hussein Ahmed, Ehsan M. W. El-Sabaa, Aliaa A. Mosa, Ahmed Shawkat Abdelmohsen, Abdelmajeed M. Moussa, Eman H. Salama, Sahar Aboulfotuh, Ahmed M. Ashmawy, Ahmed Ismail Seddik, Ibrahim M. Sayed, Haidi Karam-Allah Ramadan

**Affiliations:** 1 Department of Medical Microbiology and Immunology, Faculty of Medicine, Assiut University, Assiut 71515, Egypt; 2 Gilbert and Rose-Marie Chagoury School of Medicine, Lebanese American University, Byblos P.O. Box 36, Lebanon; 3 Molecular Biology Researches & Studies Institute (MBRSI), Assiut University, Assiut 71515, Egypt; 4 Microbiology and Immunology Department, Faculty of Pharmacy, Assiut University, Assiut 71515, Egypt; 5 Department of Medical Biochemistry, Faculty of Medicine, Assiut University, Assiut 71515, Egypt; 6 Department of Tropical Medicine and Gastroenterology, Faculty of Medicine, Assiut University, Assiut 71515, Egypt; 7 Department of Tropical Medicine and Gastroenterology, Faculty of Medicine, Aswan University, Aswan 81528, Egypt; 8 Department of Clinical Pathology, Faculty of Medicine, Sohag University, Sohag 82524, Egypt; 9 Department of Internal Medicine, Gastroenterology and Hepatology Unit, Faculty of Medicine, Assiut University, Assiut 71515, Egypt; 10 Pediatric Department, Faculty of Medicine, Aswan University, Aswan 81528, Egypt

**Keywords:** HAV, HEV, co-infection, outcome, acute liver failure

## Abstract

Hepatitis A virus (HAV) and Hepatitis E virus (HEV) are transmitted through the fecal–oral route. HAV outbreaks and one HEV outbreak have been reported in Egypt. However, the impact of HAV–HEV co-infection is not known. In this study, we assessed HEV markers in acute HAV-infected patients (*n* = 57) enrolled in Assiut University hospitals. We found that 36.8% of HAV-infected patients were also positive for HEV markers (anti-HEV IgM and HEV RNA), while 63.2% of the patients were HAV mono-infected. Demographic and clinical criteria were comparable in both HAV mono-infected patients and HAV–HEV co-infected patients. Although liver enzymes were not significantly different between the two groups, liver transaminases were higher in the co-infected patients. Six patients developed acute liver failure (ALF); five of them were HAV–HEV-co-infected patients. The relative risk of ALF development was 8.5 times higher in HAV–HEV co-infection compared to mono-infection. Three cases of ALF caused by HAV–HEV co-infection were reported in children (below 18 years) and two cases were reported in adults. All patients developed jaundice, coagulopathy, and encephalopathy; all were living in rural communities. In conclusion: HAV–HEV co-infection can be complicated by ALF. The risk of ALF development in HAV-infected patients is higher when coinfection with HEV is present.

## 1. Introduction

Hepatitis A virus (HAV) and hepatitis E virus (HEV) are the major causes of acute viral hepatitis in Egypt [1]. An analysis of acute viral hepatitis patients between 2014–2017 in Egypt revealed that 93% of the infection was related to HAV, while the incidences of HBV and HCV infection were around 2.8–3% [2]. The prevalence of HBV and HCV infection among Egyptians was reduced after the implementation of national HBV immunization for infants and by the treatment of HCV-infected patients with direct antiviral agents (DAAs) [1]. One possible cause of the increasing HAV infection among Egyptians is the absence of a national HAV vaccine program [1]. Although HEV infection is not documented in hospitals, a recent study showed that HEV is circulating among humans and animals in Egypt [3].

HAV and HEV share common features. Both viruses are enterically transmitted and present in two forms: an enveloped form in the blood and a non-enveloped form in the stool [4,5]. Outbreaks caused by HEV or HAV are mainly transmitted by the fecal–oral route or food-borne infection [6,7,8,9]. Both viruses can be transmitted through the ingestion of contaminated fruits, vegetables, and/or seafood [10]. In addition, water-borne outbreaks are caused by both viruses, since they can survive in environmental conditions for extended periods [9,11]. The genome structure of both viruses has some analogies [12,13]. The viral genome is a positive-sense single-strand RNA, with a poly-A tail at 3′, while a cap is present at 5′ of HEV, but not in the HAV genome [12,13,14]. At 5′ end of the HAV genome, there is a 450-nucleotide sequence (internal ribosome entry site (IRES)) that binds with eukaryotic initiation factors and mediates viral protein synthesis [12,15]. The genome of HAV encodes one large open reading frame (ORF), while HEV encodes three ORFs [12,15,16]. 

HAV and HEV coinfection was reported in developing countries such as India [17], Mexico [18], Egypt [19], and Venezuela [20], but it is not common in developed countries [8,21,22]. Drug use, homelessness, unavailability of the HAV vaccine, blood donation, and immunosuppression could be risk factors for HAV co-infection with HEV or other viral hepatitis [23,24,25,26]. The outcomes of HAV–HEV co-infection are not well explored. HEV diagnosis is not routinely performed in several developing countries and there is no common in vitro or in vivo model system to study this co-infection [8]. In Egypt, although a few research studies have shown that HAV–HEV infections are recorded among acute hepatitis patients [27,28,29], the outcomes of these cases were not monitored. 

In this study, we aimed to evaluate the impact of HEV–HAV co-infection among acute hepatitis patients in Egypt. We also compared the liver function profiles between patients with HAV mono-infection and patients with HAV–HEV co-infection. In addition, we monitored the outcomes of mono- and co-infections. 

## 2. Materials and Methods

### 2.1. Patients

The study included acute HAV-infected patients (*n* = 57) who either attended the outpatient clinics or were admitted to Assiut University Hospitals from July 2022 to July 2023. The enrolled patients developed one or more of the acute hepatitis symptoms such as jaundice, nausea, vomiting, abdominal pain, etc. Blood samples were collected from these patients and assessment of liver function profiles and viral markers was performed. Liver function profiles comprised the assessment of liver enzymes, including aspartate transaminase (AST), alanine transaminase (ALT), alkaline phosphatase (ALP), total bilirubin, direct bilirubin, albumin level, prothrombin time, and international normalized ratio (INR). HAV was confirmed in these patients by the detection of anti-HAV IgM (General Biologicals Corporations, Taiwan, China) by ELISA and HAV RNA as described below. Patients who tested positive for one or more markers for autoimmune hepatitis, such as anti-nuclear antibody and/or anti-smooth muscle antibody, were also not included in the study. A self-limiting course was defined as symptom resolution and patient recovery with supportive therapies within 1 to 4 weeks [30,31]. Acute liver failure (ALF) was defined as acute liver injury (elevation of liver transaminases and bilirubin), coagulopathy (INR ≥ 1.5), and changes in patient’s consciousness due to hepatic encephalopathy described by the European Association for the Study of the Liver (EASL) [31]. Recovery is defined by both the complete improvement of clinical symptoms, such as fever, jaundice, nausea, and abdominal pain, and the normalization of liver enzymes. Liver morphology by ultrasound was not included in the definition of recovery. Patients’ demographic data were collected and correlated with their laboratory and clinical parameters. A comparison between the two groups (HAV mono-infection vs. HAV–HEV co-infection) in demographics, clinical symptoms, liver functions, and outcomes of infection was performed. Patients who tested positive for HCV, HBV, EBV, or CMV were excluded from the study. 

Patients’ informed consent was obtained from the patients enrolled in the study. Pediatric patients’ consent was obtained from their parents. The research proposal was reviewed and approved by the Institutional Review Board of the Faculty of Medicine, Assiut University (IRB number: 17300764), which was performed in accordance with the Declaration of Helsinki. 

### 2.2. HEV Diagnosis and Molecular Assessment of HAV and HEV Viruses

HAV-infected patients were screened for HEV by testing anti-HEV IgM (abia HEV IgM, B Diagnostic Systems GmbH, Berlin, Germany) using ELISA as suggested by the manufacturer’s guidelines. RNA was extracted from patients’ plasma samples using the QIAamp Viral RNA Mini Kit (Qiagen, Hilden, Germany) as suggested by the manufacturer’s guidelines. Complementary DNA (cDNA) was synthesized from the viral RNA using a High-Capacity cDNA Reverse Transcription Kit (ThermoFisher Scientific, Waltham, MA, USA) following the manufacturer’s suggestions. Reverse transcription-quantitative polymerase chain reaction (RT-qPCR) was performed using virus-specific primers. The protocol performed was a two-step RT-qPCR for molecular detection of HAV and HEV. The following primer sequences were used for molecular assessment of HAV, as previously described by Jothikumar and colleagues [32]: HAV-forward primer, 5′-ggtaggctacgggtgaaac-3′; HAV-reverse primer, 5′-aacaactcaccaatatccgc-3′; and HAV-specific probe, 5′-CTTAGGCTAATACTTCTATGAAGAGATGC-3′. The primer and probe used target the 5-untranslated region (UTR) of the hepatitis A virus (HAV) and these primers could detect HAV genotypes IA, IB, IIA, IIB, III, and V, and the product size was 89 bp [32]. The following primer sequences were used for molecular assessment of HEV, as previously described by Jothikumar and colleagues [33]: HEV-forward primer, 5′-GGTGGTTTCTGGGGTGAC-3′; HEV-reverse primer, 5′-AGGGGTTGGTTGGATGAA-3′; and HEV specific probe, 5′ -TGATTCTCAGCCCTTCGC-3′. The probes used were labeled with fluorescent dye FAM at the 5′ end and with fluorescent quencher TAMRA at the 3′ end. These primers target an HEV ORF2/3 overlapping region that is conserved among HEV genotypes. The product size is 70 bp. HEV diagnosis was confirmed in the patients by both ELISA and molecular assays.

### 2.3. Sequencing of HAV and HEV in Patient Samples

To determine HAV genotypes, we performed nested PCR reactions followed by Sanger sequencing. We used primers targeting the VP1-P2A junction region as described before, with slight modification [34,35]. The first PCR reaction was performed using the following primers: forward, 5′-ACRGATTCYACATTTGGATTGGT-3′; reverse, primer 5′-CCATTTCAAGAGTCCACACACT-3′. The second PCR reaction was performed using the following primers: forward, 5′- CTATTCAGATTGCAAATTACAAT-3′; reverse, primer 5′-AAYTTCATTATTTCATGCTCCT-3′, where Y is C or T and R is A or G. The second (nested) PCR product was purified, sequenced by Sanger method, and typed using HAVNET (https://www.rivm.nl/en/havnet (accessed on 1 July 2023)). The sequenced PCR product size was trimmed off to 315 bp.

Sequencing of HEV was also performed using Sanger sequencing as previously described [36,37]. Briefly, it is a nested PCR reaction using primers targeting HEV ORF2. The product size was 348 bp. The first PCR reaction was performed using the following primers: forward, 5′-AATTATGCYCAGTAYCGRGTTG-3′; reverse, 5′-CCCTTRTCYTGCTGMGCATTCTC-3′, where (Y = C or T, R = G or A, M = A or C). The second PCR reaction was performed using the first PCR product and the following primers: forward, 5′-GTWATGCTYTGCATWCATGGCT-3′; reverse, 5′-AGCCGACGAAATCAATTCTGTC-3, where (W = A or T and Y = C or T). The sequences were analyzed using HEVnet (https://www.rivm.nl/en/hevnet (accessed on 1 July 2023), where the phylogenetic analysis was determined by alignment of the sequences with complete standard HEV sequences as determined by Smith et al. [38,39,40,41].

### 2.4. Statistics

Data from the current study were processed using GraphPad Prism software 9 (La Jolla, CA, USA). Patients’ data, including gender, symptoms, residence, age distributions, and outcomes, were presented as numbers and percentages. Patients’ ages were presented as median with minimum–maximum (range) or median with interquartile range (IQR) as specified. Liver function profiles were presented as median with IQR. *P* value was determined using a two-tailed *t*-test or Mann–Whitney test as appropriate for quantitative variables and a Chi-square test and Fisher’s exact test for categorical variables. A *p*-value of less than 0.05 was considered significant.

## 3. Results

### 3.1. Characteristics of HAV-Infected Patients Enrolled in the Study

This study included HAV-infected patients (*n* = 57); the diagnosis of HAV is confirmed by both serological and molecular assays. Most of the patients were between the ages of 10–20 years (37%), followed by patients less than 10 years old or in the range of 20–30 years old. Thirty-three patients (57.9%) were male; the remaining patients were female. Most of the patients were residing in rural areas (86%). The patients developed symptoms of acute hepatitis; jaundice was the most common symptom reported (82%), followed by vomiting (56%). The mean values (±standard deviation) of liver transaminases in those patients were 1133 (±882) IU/L and 1530 (±1070) IU/L for aspartate transaminase and alanine transaminase, respectively. The mean level of alkaline phosphatase was 201 (±62) IU/L, and the patients had elevated total bilirubin (mean 6.2 mg/dL). The albumin level was in the normal range (Table 1).

### 3.2. The Prevalence of HEV Infection among HAV-Infected Patients and Characteristics of HAV–HEV Co-Infected Patients

**Figure 1:**
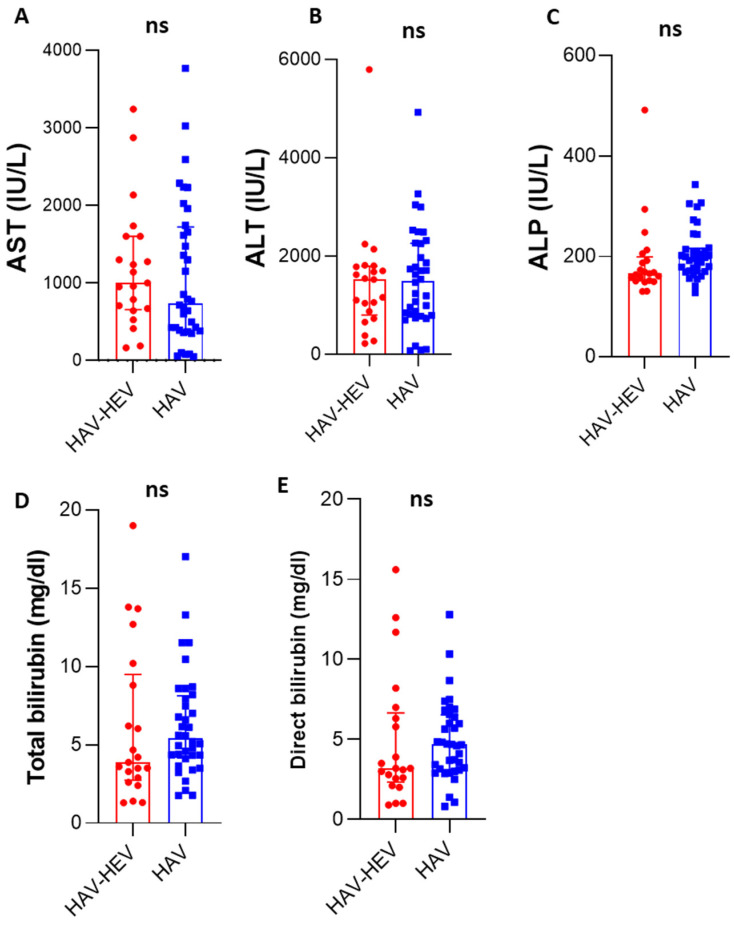
Liver function profiles of HAV–HEV coinfected patients and HAV-mono-infected patients: Liver enzymes including AST (**A**), ALT (**B**), and ALP (**C**) were compared between co-infected and mono-infected patients. (**D**,**E**) The level of total bilirubin (mg/dL) and direct bilirubin were assessed in both groups. ns, non-significant (the *p*-value was more than 0.05 as determined by the Mann–Whitney test). The red color represents the HAV–HEV co-infected group and the blue color represents the HAV-mono-infected group. All curves represent median and interquartile ranges.

Among fifty-seven HAV-infected patients, twenty-one patients were positive for active acute HEV infection (21/57, 36.8% named HAV–HEV co-infected patients), and thirty-six patients (63.2%) were only positive for HAV markers (HAV mono-infection). About 43% of HAV-HEV co-infected patients were 10–20 years old, and 29% of the patients were less than 10 years old. The mean ages of HAV-mono-infected patients (17.5 years) and HAV-HEV co-infected patients (16 years) were comparable (Table 2), and there were no significant differences in age distribution in both groups. Similarly, there was no difference in both groups in terms of gender; almost 58% of the patients in both groups were male. The median of liver transaminase (AST) in HAV–HEV co-infected patients and HAV-mono-infected patients were 1000 IU/L and 738 IU/L, respectively. The levels of total and direct bilirubin in the HAV-infected group were 5.4 mg/dL and 4.67 mg/dL, respectively, and the levels of these markers in the co-infected group were 3.88 mg/dL and 3.2 mg/dL, respectively (Figure 1). The albumin, ALT, and ALP levels were comparable in both groups. Collectively, there were no significant differences in liver function tests between both groups. Likewise, the clinical manifestations were similar in both groups. Jaundice was reported more in the HAV mono-infected patients, and vomiting and abdominal pain were reported more in co-infected patients. All co-infected patients except one were from rural areas. We performed sequencing for the samples from HAV mono-infected and HAV–HEV coinfection patients, and HAV sub-genotype IB and HEV genotype 1 subtype 1e were the viruses detected in the sequenced samples. 

### 3.3. Outcomes of HAV Mono-Infection and HAV–HEV Co-Infections and Characteristics of ALF Cases in Both Groups

**Figure 2:**
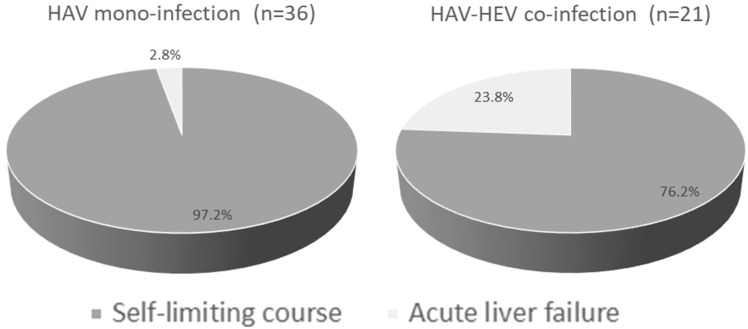
Outcomes of HAV–HEV coinfection and HAV-mono-infection. Pie chart showing the percentage of self-limiting disease and acute liver failure developed in each group. Dark grey represents an acute self-limiting course and light grey represents acute liver failure.

All HAV-mono-infected patients except one developed an acute self-limiting course (97.2%) (Figure 2). The patient who developed ALF was a 26-year-old male, who developed jaundice, fever, coagulopathy (prothrombin time 22.5 s, INR 1.98), and dark urine. The patient was hospitalized for 17 days and then recovered (Table 3). Regarding HAV–HEV co-infection, 5 out of 21 patients (23.8%) developed ALF, while 16 patients had an acute self-limiting course (76.2%) (Figure 2). HAV–HEV co-infected patients who developed ALF were 2 males and 3 females; 3 of them were 15 years old. Those patients developed jaundice, coagulopathy (INR > 1.5), and encephalopathy, and four of them had vomiting (Table 3). All these patients were residing in rural communities. Regarding liver function tests, all patients had elevated liver transaminases (at least an eight-fold increase above the normal value) and ALT level was higher or comparable to AST, except in one patient (patient #5). None of the co-infected patients died; all recovered. At recovery, the liver enzymes returned to normal and the clinical symptoms were resolved. The relative risk of ALF development was higher in HAV–HEV coinfection (8.5, 95% CI 1.0724 to 68.5) than in HAV mono-infection. None of the ALF patients had cirrhosis, acute-on-chronic liver failure (ACLF), and/or family history of liver diseases. Regarding the encephalopathy grade, three patients developed grade I and two patients developed grade II.

## 4. Discussion

Several studies confirmed the transmission of HAV and HEV is through the ingestion of contaminated food products and/or water [42,43,44,45,46,47]. However, the outcome of this co-infection is not completely understood, and there are limited data regarding this point. In this study, we aimed to study the impact of HEV–HAV co-infection among acute hepatitis patients.

HAV and HEV are highly circulating in the Egyptian environment owing to the use contaminated water sources in irrigation and planting [48]. Several outbreaks were caused by HAV or HEV in Egypt. An HAV outbreak was reported by European tourists returning from Hurghada, Egypt [46,49]. Little is known about HEV outbreaks in Egypt. One outbreak was reported in one village located in Assiut governorate (Upper Egypt) due to waterborne infection [50].

In this study, about 60% of the infected patients were 3–20 years old. Similarly, Talaat et al. showed that the median age of HAV infection in Egypt was 9 years (IQR 4–15 years) [51]. Jaundice (82%) and nausea/vomiting (56%) were the most common symptoms reported in this cohort. Likewise, Fouad and colleagues reported that 98% of acute HAV-infected children developed yellow sclera and 64% of the patients had nausea and/or vomiting [28].

The analysis of HEV infections among HAV-infected patients showed that 36.8% already had HAV–HEV co-infection. Previous studies in Egypt showed that the prevalence of HAV–HEV co-infection among acute hepatitis children ranges from 1.5% to 26% [19,28,52,53]. Zaki and colleagues reported a strong association between HEV and HAV infections in children with acute hepatitis in Egypt [53]. HAV–HEV co-infection was also reported in adults with acute hepatitis in Egypt [29]. Although the prevalence of HAV–HEV co-infection is high in this study, comparable results were obtained from other countries. In Venezuela, HAV–HEV co-infection was reported in 31% of acute hepatitis patients [20]. In addition, co-infection was recorded in 58% of children with acute hepatitis in the west of Mexico [18], while the incidence of co-infection was less in other countries such as China (10%) [54], India (13%) [55], Kenya (1%) [56], and Bangladesh [57]. HAV sub-genotype IB and HEV genotype 1 subtype 1e were the detected viruses in the study. These two viruses were previously reported in Egypt in acute hepatitis patients, environmental samples, and during the outbreaks [49,58,59,60,61].

When comparing liver function profiles and demographic features between HAV-mono-infected patients and HAV–HEV co-infected patients, we did not find any significant differences between both groups in terms of age, gender, clinical manifestations, liver enzymes, bilirubin, and albumin level. Moreover, there were no significant differences between HAV–HEV co-infected patients and HAV-mono-infected patients in terms of liver transaminases and bilirubin. In a parallel investigation, several studies showed that there were no significant differences between co-infection of HAV/HEV and single viral infection (HAV or HEV) in terms of liver function profiles, prognosis, clinical symptoms, disease duration, death rate, etc. [62,63]. Li and colleagues showed that HAV–HEV co-infected patients had elevated levels of ALT, AST, and bilirubin compared to HAV-mono-infected or HEV-mono-infected patients, but the elevation was not significantly different [54]. Other studies showed that co-infection led to a higher elevation of liver enzymes compared to mono-infection [64,65]. The differences in the liver profiles in this study and other reports could be attributed to the differences in the patient cohort, geographical distribution, circulating viruses causing the coinfection, etc. Future studies should address these points.

In this study, HAV–HEV co-infection led to a severe disease outcome, and about five patients (24%) developed acute liver failure (ALF), while only one patient with HAV infection (2.8%) had ALF. Likewise, Fouad et al. reported that none of the HAV-infected children in Egypt progressed to fulminant hepatic failure [28]. In addition, Manka and colleagues reported that HAV infection rarely progressed to ALF [66], while HEV infection could progress to ALF [67]. This explains the high rate of ALF with co-infection, but not with HAV mono-infection. Arora and colleagues reported that 20% of ALF in children was caused by HAV–HEV co-infection, followed by HEV mono-infection (13%), and then HAV mono-infection (9%) [17]. The five co-infected patients in this study developed coagulopathy, jaundice, and encephalopathy. Similarly, several reports and case studies showed that HAV–HEV coinfection could be complicated by encephalopathy, jaundice, cholecystitis, and fulminant hepatic failure, which could eventually to death [17,64,68]. 

In this study, most of the HAV–HEV co-infected patients were from rural communities. The risk of infection is higher in rural communities than in urban communities due to the lower level of hygiene and educational background of villagers and frequent contact with animals that probably live inside the same home. Previous studies in Assiut governorate showed that HEV infections and HAV–HEV co-infections were reported in patients resident in villages and rural regions [27,58]. In addition, recent studies showed the circulation of HEV among ruminants in the villages of Assiut governorate and the presence of the HEV virus in the milk and liver of infected animals [37,69]. 

The current study has both advantages and limitations. To our knowledge, it is the first report to study the impact of HAV–HEV co-infection in Upper Egypt in children and adults. It highlights the possibility of the progression of this co-infection to ALF and therefore the clinicians should be aware of the disease outcomes. Moreover, the study underscores the importance of the HAV vaccine and the application of preventative measures to reduce the risk of HAV and/or co-infections. In addition, we recommend the assessment of HEV diagnosis for all cases of acute hepatitis caused by HAV to characterize the co-infection and implement early strategies to reduce the risk of complications. The following limitations should be considered. First, the study could not explain the molecular aspects of ALF caused by HAV–HEV co-infection due to the lack of liver biopsies from those patients. Moreover, the study did not include HEV-mono-infected patients; however, our observations from previous studies, including HEV mono-infection in the same area, showed that liver transaminases were lower in HEV mono-infection than in HAV-mono-infection and/or HAV–HEV co-infection [58,70]. In addition, the number of enrolled patients was not large enough to draw a robust conclusion, and this could explain the reason that there were no significant differences between HAV mono-infection and HAV–HEV co-infection. Further studies are needed to confirm these findings.

## 5. Conclusions

HAV–HEV co-infection is reported among acute hepatitis patients, especially those living in rural communities. The co-infected patients could progress to ALF. Implementation of a HAV vaccine program could reduce the complications of HAV infection and/or coinfection of HAV with other viral hepatitis.

## Figures and Tables

**Table 1:** Characteristics of HAV-infected patients enrolled in the study.

Items	HAV Patients (*n* = 57)
Age (years)	15 (3–62)
(Median, minimum–maximum)	
<10 years	15/57 (26.3%)
10–20 years	21/57 (36.84%)
20–30 years	15/57 (26.3%)
>30 years	6/57 (10.52%)
Gender	
Male	33/ 57 (57.9%)
Female *	24/57 (42.1%)
Residence	
Rural	49/57 (86%)
Urban	8/57 (14%)
Clinical presentation	
Jaundice	47/57 (82.46%)
Nausea/Vomiting	32/57 (56.14%)
Fever	22/57 (38.6%)
Abdominal pain	20/57 (35.08%)
Liver function tests	
(Median, IQR)	
AST (IU/L)	850 (424–1635)
ALT (IU/L)	1521 (803–1918)
ALP (IU/L)	187 (162–212)
Total bilirubin (mg/dL)	4.95 (3.5–8.4)
Direct bilirubin (mg/dL)	3.9 (2.9–6.5)
Serum albumin g/dL	3.9 (3.31–4.1)

* None of the enrolled patients were pregnant women.

**Table 2:** Comparison between demographic and laboratory parameters of HAV mono-infection and HAV–HEV co-infection.

Items	HAV-Mono-Infection (*n* = 36)	HAV-HEV Co-Infection (*n* = 21)	*p* Value
Age (years)	15 (8.5–27)	15 (7–21)	ns
(Median, IQR)			
<10 years	9/36 (25%)	6/21 (28.6%)	
10–20 years	12/36 (33.3%)	9/21 (42.9%)	
20–30 years	10/36 (27.8%)	5/21 (23.8%)	
>30 years	5/36 (13.9%)	1/21 (4.8%)	
Gender			ns
Male	21/36 (58.3%)	12/ 21 (57.9%)	
Female	15/36 (41.7%)	9/21 (42.1%)	
Residence			ns
Rural	29/36 (80.6%)	20/21 (95.2%)	
Urban	7/36 (19.4%)	1/21 (4.8%)	
Clinical presentation			
Jaundice	32/36 (88.9%)	15/21 (71.4%)	ns
Nausea/Vomiting	19/36 (52.78%)	13/21 (61.9%)	ns
Fever	14/36 (39%)	8/21 (38%)	ns
Abdominal pain	11/36 (30.6%)	9/21 (42.9%)	ns
Hepatitis markers			
Anti-HAV IgM	36/36 (100%)	21/21 (100%)	
HAV RNA	36/36 (100%)	21/21 (100%)	
Anti-HEV IgM	0/36 (0%)	21/21 (100%)	
HEV-RNA	0/36 (0%)	21/21 (100%)	
Outcome			0.02 (*)
Self-limiting course	35/36 (97.2%)	16/21 (76.2%)	
Acute liver failure	1/36 (2.8%)	5/21 (23.8%)	
Encephlaopathy	0/36 (0%)	5/21 (23.8%)	0.0049 (**)

ns: not significant. *, **: *p* values are less than 0.05, 0.01, respectively.

**Table 3:** Characteristics of ALF cases caused by HAV mono-infection and HAV–HEV co-infections.

Items	HAV Mono-Infection	HAV–HEV Co-Infection				
		Patient #1	Patient #2	Patient #3	Patient #4	Patient #5
Age (years)	26	15	15	15	62	21
Gender	male	male	male	female	female	female
Residence	Urban	Rural	Rural	Rural	Rural	Rural
Clinical symptoms	Jaundice, fever, and dark urine	Jaundice, fever, vomiting, dark urine, abdominal pain, and encephalopathy	Jaundice, vomiting, abdominal pain, and encephalopathy	Jaundice, fever, vomiting, and encephalopathy	Jaundice, dark urine, and encephalopathy	Jaundice, vomiting, and encephalopathy
Liver function tests						
AST (IU/L)	713	2134	707	959	1000	1141
ALT (IU/L)	1071	2140	1053	1159	1800	379
ALP (IU/L)	245	159	294	169	158	131
Total bilirubin (mg/dL)	8.2	3.3	2.6	13.7	13.8	8.8
Direct bilirubin (mg/dL)	6.4	2.6	2.0	12.6	11.7	7.0
Serum albumin g/dL	3.1	4.4	4.0	2.7	2.6	2.8
International normalized ratio (INR)	2.0	2.6	2.0	1.9	1.8	2.5
Recovary (Days)	17	14	16	17	27	18

## Data Availability

The study data are present in the main text, and for further inquiries please contact the corresponding authors.
